# Seroprevalence and associated risk factors of strongyloidiasis in indigenous communities and healthcare professionals from Brazil

**DOI:** 10.1371/journal.pntd.0011283

**Published:** 2023-04-27

**Authors:** Vamilton Alvares Santarém, Fernando Rodrigo Doline, João Henrique Farinhas dos Santos, Isabella Braghin Ferreira, Bruna Barroso Gomes, Dirce Mary Correa Meisel, Leandro Meneguelli Biondo, Susana Angélica Zevallos Lescano, Ronaldo Cesar Borges Gryschek, Rogério Giuffrida, Andrea Pires dos Santos, Louise Bach Kmetiuk, Fabiana Martins de Paula, Alexander Welker Biondo

**Affiliations:** 1 Graduate College in Animal Sciences, University of Western São Paulo (UNOESTE), Presidente Prudente, Brazil; 2 Graduate College of Cell and Molecular Biology, Federal University of Paraná (UFPR), Curitiba, Brazil; 3 Laboratory of Medical Investigation, Clinical Hospital of the University of São Paulo, São Paolo, Brazil; 4 National Institute of the Atlantic Forest (INMA), Brazilian Ministry of Science, Technology, and Innovation, Santa Teresa, Espirito Santo, Brazil; 5 Department of Comparative Pathobiology, Purdue University, West Lafayette, Indiana, United States of America; Federation University Australia, AUSTRALIA

## Abstract

*Strongyloides stercoralis*, a pathogenic roundworm, is considered endemic in several tropical and subtropical areas worldwide. Indigenous populations have the highest soil-transmitted helminthiases-related mortality rates, but the prevalence and risk factors associated with *S*. *stercoralis* in Brazilian indigenous populations have not been established. Thus, the present study aimed to assess the seroprevalence and associated risk factors for *S*. *stercoralis* in indigenous communities and the healthcare professionals serving them in Brazil. Indigenous populations living in nine communities and healthcare professionals were tested for anti- *S*. *stercoralis* antibodies by ELISA. A questionnaire was used to assess socio-epidemiological information. Associated risk factors for seropositivity were tested by chi-square or Fisher’s exact tests, using univariate analyses and multivariate logistic regression. Overall, 174/463 (37.6%; CI 95%: 33.3–42.1) indigenous persons and 77/147 (52.4%; 95% CI: 44.3–60.3) healthcare professionals were seropositive for anti- *S*. *stercoralis* antibodies. Seropositivity among the two groups was statistically significant (p = 0.0016; OR = 0.547; 95% CI: 0.376–0.796) and revealed that healthcare professionals were 1.83 times more likely to be seropositive. The multivariate analysis showed that being male or being adult were also risk factors, while having a septic tank as a sanitary facility represented a protective factor for *S*. *stercoralis* exposure in indigenous persons. None of the variables evaluated were associated with *S*. *stercoralis* exposure in the professional group. The study herein has reported a high seroprevalence to *Strongyloides stercoralis* in indigenous communities of Brazil and healthcare professionals, warning for potential public health concerns of strongyloidiasis in such populations.

## Author summary

Human strongyloidiasis, caused mainly by nematoda *Strongyloides stercoralis*, with estimate of infecting around 613.9 million people worldwide, and may play a pivotal role in causing morbidity in aboriginals and indigenous populations worldwide. Nevertheless, prevalence and risk factors for *S. stercoralis* in Brazilian indigenous populations remains to be fully established. The present study was the first comparative report of *S*. *stercoralis* seroprevalence in indigenous population and healthcare professionals. A high seroprevalence to *S. stercoralis* in indigenous communities of Brazil along with even higher exposure of healthcare professionals was reported and should be considered as vulnerability and health worker exposure. Male and adult indigenous persons were more likely seropositive as associated risk factors, while having septic tank as a sanitary facility represented a protective factor for *S*. *stercoralis* exposure. Thus, the present study has alerted for the public health concerns of strongyloidiasis in such vulnerable populations, particularly when lacking sanitation conditions. Finally, a further One Health approach may contribute for better understanding the strongyloidiasis exposure including the environmental, animal, and human components to battle this important parasitic neglected disease in indigenous communities.

## Introduction

Although underestimated, human strongyloidiasis, caused mainly by nematoda *Strongyloides stercoralis*, with yearly estimate of infection around 613.9 million people worldwide [1,2]. A recent ecological niche model study estimated that more than 2.6 billion persons may be at risk for infection by *S*. *stercoralis* [3]. Strongyloidiasis is asymptomatic or oligosymptomatic in most infected individuals [4]. Dysregulation of cellular immune response, however, may trigger the reactivation of larvae and result in a life-threatening hyper-infection syndrome affecting multiple organ systems [5].

As important, reactivation of infection has been observed in individuals under continuous immunosuppressive treatment such as chemotherapy [6] or corticosteroids [7], and those suffering from chronic alcoholism [8], or immunosuppressed [9,10]. Reactivation risk, hyper-infection syndrome development, and disseminated disease have also been observed in persons with tissue transplants of both solid organs and hematopoietic stem cells [11–13].

The potential for autoinfection and parthenogenetic reproduction within the host is considered particular to *S*. *stercoralis* [14] and results in persistent infections lasting decades in endemic areas [15]. Prevalence in adults is higher than in children, possibly due to continuing exposure with age [16,17]. Cumulative exposure and autoinfection cycle favor the perpetuation of infection [18], particularly in areas with low hygiene and socioeconomic standards [19].

Strongyloidiasis may play a pivotal role in causing morbidity in aboriginal and indigenous populations [20]. Brazil is considered to have the highest number of people at risk for *S*. *stercoralis* infections [3]. Due to poor sanitation of living conditions, Brazilian Indigenous populations are highly predisposed to infection by intestinal parasites [21,22].

A nationwide population-based study of all deaths in Brazil from 2000 to 2011 revealed indigenous populations as one of the groups with the highest soil-transmitted helminths related mortality rates [23]. Nevertheless, prevalence and risk factors associated with *S*. *stercoralis* infection among Brazilian indigenous populations are not fully established. This study aimed to assess the seroprevalence and risk factors associated with having anti- *S*. *stercoralis* antibodies in the indigenous communities living in southern and southeastern Brazil. Members of the communities and their healthcare professionals were included.

## Material and methods

### Ethics statement

This study was approved by the National Research Ethics Committee of the Brazilian Ministry of Health (protocol 52039021.9.0000.0102) and by the Ethics Committee on Animal Use of the Federal University of Paraná (protocol number 033/2021).

### Study timeline and area

In the present study, serum and soil samplings and epidemiological questionnaires were obtained in nine indigenous communities of the Guarani, Terena, and Kaingang ethnic groups in the states of Paraná and São Paulo, from December 2020 through February 2022. Geographic location of indigenous communities are presented with the total and sampled populations in S1 Table.

### Study design

This was a cross-sectional study anti-*Strongyloides stercoralis* antibodies (IgG) and associated risk factors in indigenous communities of southern and southeastern Brazil. In addition, prevalence and risk factors were tested in non-indigenous healthcare professionals working in these Indigenous communities.

### Indigenous characteristics

In 2010, the Brazilian Ministry of Health created a new branch called Special Secretary of Indigenous Health (SSIH), composed of approximately 22,000 health professionals (52% indigenous) who provide local health assistance to an estimated indigenous population of 896,000 individuals (46% under 19 years old). This is approximately 0.4% of the total Brazilian population, distributed across 505 indigenous lands (12.5% of the national territory), with 305 ethnicities and 274 spoken languages [24].

The SSIH has been divided into 34 Special Districts of Indigenous Health (SDIH). The SDIH—Seashore South region of this study was managing a total of 25,784 indigenous persons with 25 ethnicities living in an area of 174,521 km^2^ (twice the size of Portugal) with 129 indigenous communities located in four states: Santa Catarina, Paraná, São Paulo, and Rio de Janeiro. According to the SSIH administration, indigenous populations underwent a preventive deworming program of Albendazole (children 6–14 years old: 400 mg SID for 5 days; >14 years old: 400 mg in single dose),twice per year (May and November) to mitigate the risks of helminth infection. The Program was implemented in 2017 (May 30th, 2017).

The socioeconomic characteristics of indigenous communities of Paraná and São Paulo states are distinct. Communities of Paraná state had an indigenous population with strong environmental ties that relied on natural resources for living, such as wildlife hunting, fishing, and a small amount of subsistence agriculture for their own community [25,26]. The communities of Paraná lacked water treatment systems and had no septic tanks in their households. Communities of São Paulo state relied on agriculture (the most relevant regional economic activity), cultivating crops for both internal consumption and external trading [27]. Persons in these indigenous communities also worked at the nearby rural farms and urban areas and had low hunting activity [28]. Handicrafts also represented a source of subsistence for some families in these communities [29]. The indigenous communities of São Paulo had artesian wells for water supply and septic tanks for feces disposal.

### Blood collection

The expected prevalence of Strongyloidiasis in Brazil was estimated between 10 to 20% according to a systematic review study [30]. Thus, the minimum sample size was determined with the following parameters: expected prevalence of 15%, confidence level of 95%, sampling error around the estimated proportion of 5% for an infinite population (> 25,000 indigenous people), concluding that a minimum of 196 individuals are needed [31]. In order to provide greater confidence to the estimates, we expanded the collection of samples, aiming to obtain a greater representativeness of the population with the inclusion of indigenous people independent on age groups and genders.

The indigenous participants were sampled in communities, after signing a consent form and completing an epidemiological questionnaire. Blood samples (10 mL) were then individually collected by cephalic venipuncture performed by certified nurses, physicians, and pharmacists. The samples were collected into a tube with serum separator gel and centrifuged at 800g for five minutes after clot formation; then, serum was removed and stored at -20°C until testing.

Healthcare professional samplings were taken during incursions at the indigenous communities, and also in specific visits to the Special Department of Indigenous Health (SDIH) regional headquarters—“SDIH—Seashore South”, one of the 34 nationwide divisions belonging to the Special Secretary of Indigenous Health, Brazilian Ministry of Health. Groups were categorized according to their roles and level/frequency of contact during visits to the indigenous populations. These included: 1) high-level contact with professionals (physicians, nurses, nursing technicians, drivers, and teachers) visiting frequently; 2) medium-level contact with groups of the multi-disciplinary SDIH professionals visiting periodically; and 3) low-level contact with administration and health professionals visiting sporadically.

### Data collection

Evaluation of the epidemiological characteristics of the indigenous people was based on a questionnaire that assessed potential risk for strongyloidiasis (S2 Table). Epidemiological assessment was made by interview with individual questionnaires during blood samplings, applied by trained health professional, with the assistance of an indigenous healthcare worker translator when necessary.

### Laboratory testing of samples

Two types of antigenic extract were made. The first was infective larvae (iL3) from *Strongyloides venezuelensis* obtained from 48-hour charcoal fecal cultures of male Wistar rats (*Rattus norvegicus*) infected experimentally (Animal Research Ethics Committee of FMUSP, protocol No. 0356A). The iL3 were recovered using a modified Baermann technique: the larvae were treated with 0.25% sodium hypochlorite for 5 minutes, then washed four times with sterile distilled water and centrifuged at 4,000 x g for one minute, and then stored at -20°C until use [32]. The second was made from surface cuticle from *S*. *venezuelensis* iL3 and was extracted with CTAB detergent. Briefly, approximately 200 mg (dry weight) of iL3 were resuspended in 1 mL of 0.01 M phosphate-buffered saline, pH 7.2 (PBS) containing 0.25% CTAB detergent, and incubated overnight at 4°C. The suspension was centrifuged at 12,400 x g for 10 minutes, and the supernatant was stored at -20°C. This antigenic extract was named L3-CTAB. The protein content from both types of antigenic extracts was quantified by the commercial kit Pierce BCA Protein Assay Kit (Thermo Fisher Scientific, Waltham, MA, USA).

### Extraction of surface cuticle from *S*. *venezuelensis* iL3 with CTAB detergent (L3-CTAB)

*S*. *venezuelensis* iL3 were obtained from 48-hour charcoal fecal cultures from male Wistar rats (*Rattus norvegicus*) experimentally infected (Animal Research Ethics Committee of FMUSP, protocol N° 0356A). These iL3 were recovered by using a modified Baermann technique and then stored at -20°C until use (32). Briefly, approximately 200 mg (dry weight) of iL3 were resuspended in 1 mL of phosphate-buffered saline 0.01 M, pH 7.2 (PBS) containing 0.25% CTAB detergent and incubated overnight at 4°C. The suspension was centrifuged at 12,400 x g for 10 minutes and the supernatant was stored at -20°C. The protein content was quantified by the commercial kit Pierce BCA Protein Assay Kit (Thermo Fisher Scientific, Waltham, MA, USA).

### Enzyme linked immuno sorbent assay

ELISA for detection of IgG antibodies was based on an established protocol used for research and daily routine use at the Clinical Hospital, School of Medicine, University of São Paulo [33]. Briefly, commercial 96-well flat bottom microtitration polystyrene plates (Costar, Sigma-Aldrich Chemical Co., St. Louis, MO, USA) were coated (100 μL/well) with the antigenic extracts at a concentration of 10 μg/mL, diluted in carbonate-bicarbonate buffer (0.06 M, pH 9.6), and incubated overnight at 4°C. After three washings (300 μL/well) of 5 min each with PBS containing 0.05% Tween-20 (PBS-T), the plates were blocked with 5% non-fat milk diluted in PBS-T for 2 hours at 37°C. The plates were then washed with PBS-T as described above, and serum samples tested in duplicate (100 μL/well) at 1:200 dilution in blocking solution, and incubated for 45 min at 37°C. After three washings with PBS-T, peroxidase-conjugated goat anti-human IgG (Fc specific) antibodies (Sigma-Aldrich Chemical Co., St. Louis, MO, USA) were diluted at 1:10,000 in blocking solution, added to the plates (100 μL/well) and incubated for 45 min at 37°C. After three washings with PBS-T, tetramethylbenzidine chromogen solution (Thermo Fischer Scientific, Waltham, MA, USA) was added (100 μL/well) and incubated for 7 min at 37°C; the reaction was stopped with 2N sulfuric acid (50 μL/well). The plates were finally read at 450 nm using an ELISA reader (Thermo Fischer Scientific, Waltham, MA, USA) and results expressed in optical density (OD) units.

The ELISA used herein has shown 95.0% sensitivity and 97.8% specificity, and 0.286 cut off [33]. Diagnostic parameters were determined by the ROC curve, using serum samples that were parasitologically positive for *S*. *stercoralis*, negative, and positive for other parasites, as previously described [33]. All assays were monitored by including positive (pool of positive serum samples for *S*. *stercoralis*) and negative (pool of negative serum samples for *S*. *stercoralis*) control sera as well as a blank without any serum sample. A 10% variation in the results from positive and negative control sera was tolerated as an internal control in each assay.

All samples close to the cut-off (with values 10% above or below the cut-off value) were retested to confirm the results, with samples presenting OD values greater than and equal to 0.286 were considered as reagents. The in-house ELISA test used in the present study was developed in the Medical Investigation Laboratory, School of Medicine, University of São Paulo, Brazil. The test has been based on the heterologous extract of *S*. *venezuelensis* (CTABL3), with cut-off, sensitivity, and specificity values of 0.286, 95% and 97.8%, respectively, as already established (Gomes et al 2022) [33]. Positive serum samples for *S*. *stercoralis* and negative for other parasites, along with analysis of the ROC curve were used to ensure quality control of CTAB-L3 diagnostic parameters.

### Statistical analysis

The collected data was double-checked to exclude redundant or missing values. Continuous variables (age in years) were categorized based on quartiles of the age groups. The proportions of seropositivity for *S*. *stercoralis* of indigenous household-level and non-indigenous workers in the communities were compared by the chi-square test. Two analyses were carried out, including one with all indigenous age groups and another considering only adult indigenous people (> 18 years old), assessing the influence of children on seropositivity. To assess the risk/protective factors for seropositivity, chi-square or Fisher’s exact tests were performed using univariate analyses, considering the total indigenous population, regardless of age. Variables with *P* <0.20 in the univariate analysis were selected for the multivariate logistic regression model, including all the independent variables with *P* <0.20 in the univariate analysis. The final model had as inclusion criteria *P* <0.05. Results are shown as odds ratios (OR) and 95% confidence intervals (CI). *P*-values less than 0.05 were considered statistically significant. All statistical analyses were performed using R software [34].

## Results

### Characteristics of the study population

Regarding the indigenous population, 241 out of 463 (52.1%) were females and 222 (47.9%) were males. In total, 325 (70.3%) were adults and 137 (29.7%) under 18 years old, ranging from 3 to 89 years of age (median: 26). One indigenous person referred no knowledge of his exactly age. Majority of indigenous belonged to Guarani ethnicity (220/463; 47.51%).

### Prevalence of anti-*Strongyloides* IgG antibodies

**Fig 1:**
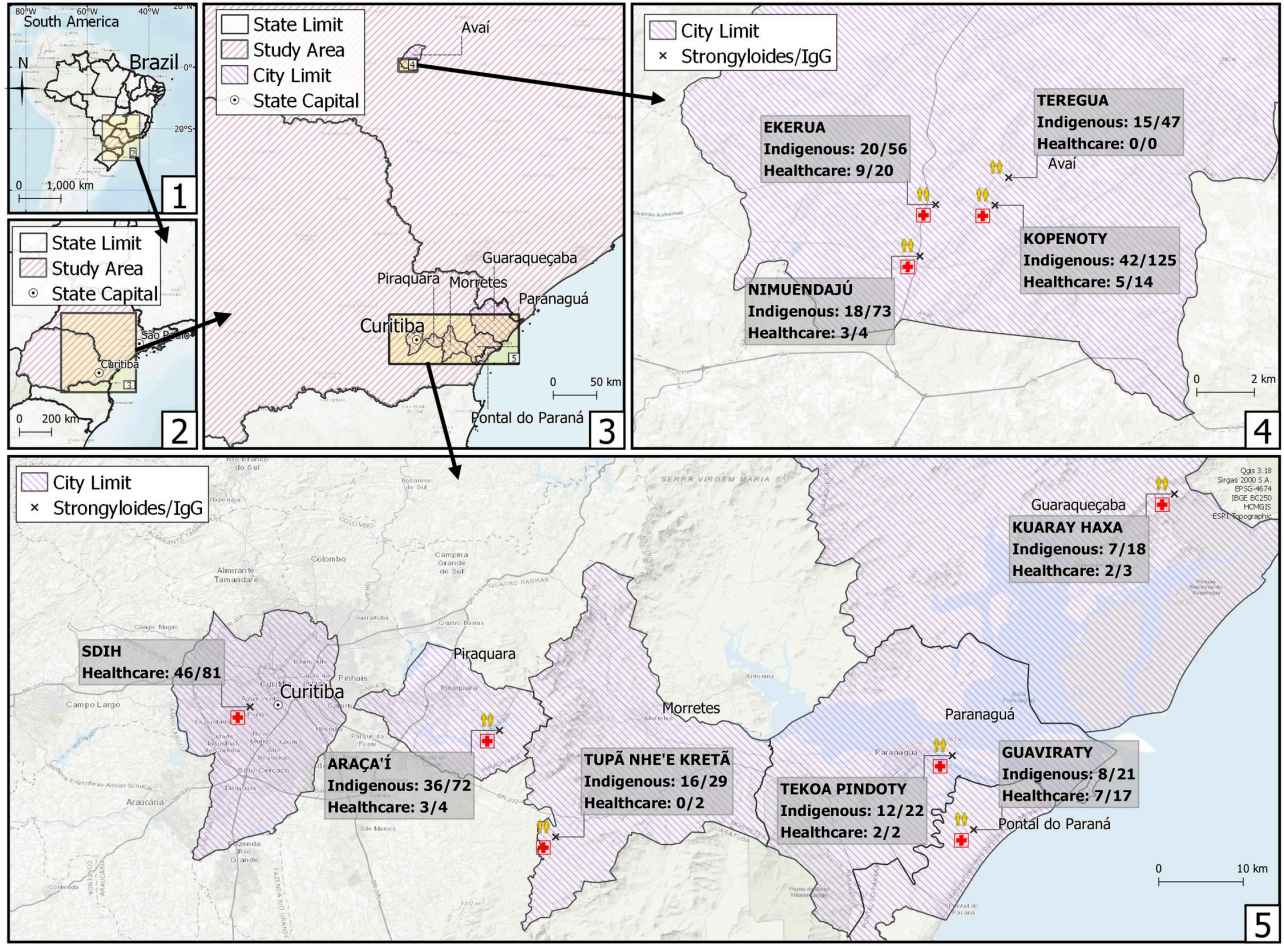
Sampling location and distribution of anti- S. stercoralis antibodies in indigenous communities of Paraná and São Paulo state, including indigenous populations and healthcare professionals. (All components of the map are open source and open data—Direct link to source of Icons and symbols used: https://github.com/qgis/QGIS/tree/master/images/svg; Direct link of the boundaries from brazilian government official public data: https://geoftp.ibge.gov.br/cartas_e_mapas/bases_cartograficas_continuas/bc250/versao2021/post_gis/bc250_2021_11_18.zip; direct link to the base layer of the map: https://maps.wikimedia.org/#8/-24.009/-48.126).

A total of 174/463 (37.6%; CI 95%: 33.3–42.1) indigenous persons were seropositive by the ELISA test detection of anti-*S*. *stercoralis* antibodies. Prevalence was significantly higher qui-square with Yates correction (12.57, df = 1, p = 0.0004) in indigenous persons living in Paraná (48.8%; 95% CI: 41.2–56.4) than in São Paulo state (31.6%, 95% CI: 26.6–37.0). The higher seroprevalence (55.2%) was observed in Tupã Nhe-e Kretã indigenous community (Fig 1 and Table 1). Regarding ethnicity, seropositivity was the most prevalent in Guarani indigenous (Table 2).

**Table 1:** Prevalence rates for anti- *S*. *stercoralis* antibodies in indigenous population living in communities in southern and southeastern Brazil.

Communities	Positive ELISA test (Anti-St)	Participants	Prevalence (%) (95% CI)
**Paraná State—South**			
Tupã Nhe’e Kretã	16	29	55.2 (42.5–77.6)
Pidoty	12	22	54.5 (34.7–73.1)
Araça’i	36	72	50.0 (38.8–61.3)
Kuaray haxa	7	18	38.9 (20.3–61.4)
Guaviraty	8	21	38.1 (20.8–59.1)
**Subtotal**	79	162	48.8 (41.2–56.4)
**São Paulo State—Southeast**			
Ekeruá	20	56	35.7 (24.5–48.8)
Kopenoti	42	125	33.6 (25.9–42.3)
Tereguá	15	47	31.9 (20.4–46.2)
Nimendaju	18	73	24.6 (16.2–35.6)
**Subtotal**	95	301	31.6 (26.6–37.0)
**Total**	174	463	37.6 (33.3–42.1)

95% CI: 95% Confidence Interval

anti-St = anti- *S*. *stercoralis* antibodies.

**Table 2:** Associated risk factors for anti- *S*. *stercoralis* antibodies in indigenous population in Brazil by univariate and multivariate statistical analysis (N = 463).

	ELISA test (Anti-*St*)		Univariate analysis		Multivariate analysis	
**Variables**	Positive (%) n = 174	Negative (%) n = 289	OR (CI 95%)	*p*-value	OR (CI 95%)	*p*-value
**Sex**				0.005		
Female	75 (43.1)	165 (57.1)	Reference			
Male	99 (56.9)	124 (42.9)	1.76 (1.20–2.57)		1.76 (1.18–2.64)	0.006
**Age, y**				0.000		
< 18	31 (17.9)	106 (36.7)	Reference			
18–26	40 (23.1)	58 (20.1)	2.35 (1.33–4.18)		2.47 (1.24–4.00)	0.008
27–40	50 (28.9)	66 (22.8)	2.58 (1.50–4.48)		2.48 (1.42–4.35)	0.002
41–89	52 (30.1)	59 (20.4)	2.99 (1.74–5.23)		3.14 (1.80–5.55)	0.000
**Ethnicity**				0.378		
Guarani	91 (52.3)	129 (44.6)	Reference			
Kaigang	4 (2.30)	7 (2.42)	0.82 (0.20–2.88)			
Nhandevá	10 (5.75)	15 (5.19)	0.95 (0.39–2.20)			
Terena	69 (39.7)	138 (47.8)	0.71 (0.48–1.05)			
**Educational level**				0.471		
Illiterate	2 (1.15)	11 (3.81)	Reference			
Primary	23 (13.2)	39 (13.5)	3.04 (0.72–22.9)			
Elementary	68 (39.1)	116 (40.1)	3.03 (0.77–21.9)			
High	62 (35.6)	99 (34.3)	3.23 (0.82–23.4)			
Graduate	19 (10.9)	24 (8.30)	4.04 (0.92–31.2)			
**Feces disposal**				0.001		
Open defecation	43 (24.7)	41 (14.2)	Reference			
Pit latrine	36 (20.7)	42 (14.5)	0.82 (0.44–1.52)		0.75 (0.39–1.43)	0.387
Septic tank	95 (54.6)	206 (71.3)	0.44 (0.27–0.69)		0.43 (0.26–0.71)	0.001
**Drinking water source**				0.001		
River	52 (29.9)	49 (17.0)	Reference			
Spring	27 (15.5)	34 (11.8)	0.75 (0.39–1.42)			
Artesian well	95 (54.6)	206 (71.3)	0.44 (0.27–0.69)			
**Dog owner**				0.397		
No	32 (18.4)	64 (22.1)	Reference			
Yes	142 (81.6)	225 (77.9)	1.26 (0.79–2.04)			

Anti-*St* = anti- *S*. *stercoralis* antibodies

Although a total of 168 healthcare professionals attending indigenous communities were sampled herein, 21/168 (12.5%) were indigenous workers living in these communities and exposed to same environmental conditions. Thus, analyses were made for only for the 147/168 (87.5%) non-indigenous healthcare professionals (ni-HP). A total of 77/147 (52.4%; 95% CI: 44.3–60.3) healthcare professionals were seropositive by the ELISA test for detection of anti- *S*. *stercoralis* antibodies. The difference in seropositivity levels among the two groups (indigenous persons vs healthcare professionals) was statistically significant (p = 0.0016; OR = 0.547; 95% CI: 0.376–0.796), and revealed that healthcare professionals were more likely seropositive (OR = 1.83). Excluding the indigenous population aging less than 18 years, the prevalence of seropositivity was similar in indigenous adults (56.1%; 183/326) and healthcare professionals (53.4%; 77/147). Nonetheless, no statistical difference was observed (p = 0.106) between these groups.

### Risk and protective factors for *S*. *stercoralis* exposure

Based on the univariate model (Table 2), the odds of seropositivty in indigenous males increased 1.75-fold (OR: 1.75; CI 95%: 1.20–2.57; p = 0.005). Results also showed that the risk of having a seropositive test was directly proportional to the age of the indigenous individuals. The presence of septic tank as a sanitary facility was a protective factor, with 56% lower odds for *S*. *stercoralis* seropositivity (OR = 0.44; CI 95%: 0.26–0.71; p = 0.001) compared with open defecation. Similarly, drinking water from artesian wells was a protective factor (OR: 0.44; CI: 0.27–0.69; p = 0.001). No association with seropositivity was observed when comparing the ethnicities or owing to not-owning dogs. It was observed an average of 2.5 dogs (range: 1 to 12 dogs) per indigenous person.

The multivariate analysis retained the same predictors observed in the univariate analysis, showing that being an adult and male were risk factors while having a septic tank sanitary facility was a protective factor for strongyloidiasis in indigenous persons. Due to the collinearity observed between the feces disposal methods and drinking water source variables, only the feces disposal methods was selected for inclusion in the multivariate analysis. The ROC curve has indicated poor to fair performance (0.68; 95% CI: 61.1–71.7) in predicting seropositivity in indigenous people. However, the approach was of limited value for the outcome interpretation of logistic regression models, given that AUC has a probabilistic interpretation (S1 Fig).

In the healthcare professional group, none of the evaluated variables were associated with seropositivity (Table 3).

**Table 3:** Associated risk factors for anti- *S*. *stercoralis* antibodies in healthcare professionals in Brazil by uni- and multivariate statistical analysis (N = 147).

	ELISA test (Anti-*St*)		Univariate analysis		Multivariate analysis	
**Variables**	Positive (%) (n = 77)	Negative (%) (n = 70)	OR (CI 95%)	*p*-value	OR (CI 95%)	*p*-value
**Sex**				0.879		
Female	46 (59.7)	40 (57.1)	Reference			
Male	31 (40.3)	30 (42.9)	0.90 (0.46–1.74)			
**Age, y**				0.266		
20–29	19 (24.7)	23 (32.9)	Reference			
30–38	21 (27.3)	12 (17.1)	2.09 (0.82–5.48)			
39–46	21 (27.3)	15 (21.4)	1.68 (0.68–4.22)			
47–65	16 (20.8)	20 (28.6)	0.97 (0.39–2.40)			
**Ethnicity**						0.147
White	68 (88.3)	67 (95.7)	Reference	0.182	Reference	
Non-white	9 (11.7)	3 (4.29)	2.84 (0.79–14.0)		2.73 (0.76–12.83)	
**Working local (State)**				0.453		
Paraná	14 (18.2)	14 (20.0)	Reference			
Paraná and São Paulo	46 (59.7)	35 (50.0)	1.31 (0.55–3.15)			
São Paulo	17 (22.1)	21 (30.0)	0.81 (0.30–2.19)			
**Contact level with indigenous persons**				0.875		
High	22 (28.6)	19 (27.1)	Reference			
Low	17 (22.1)	18 (25.7)	0.82 (0.33–2.04)			
Medium	38 (49.4)	33 (47.1)	1.00 (0.46–2.16)			
**Consumption of water in the indigenous community**						0.166
No	53 (68.8)	56 (80.0)	Reference	0.175	Reference	
Yes	24 (31.2)	14 (20.0)	1.80 (0.85–3.94)		1.72 (0.80–3.77)	
**Washing fruits and vegetables before meals**				1.0		
No	1 (1.30)	1 (1.43)	Reference			
Yes	76 (98.7)	69 (98.6)	1.10 (0.03–43.5)			
**Having meals in indigenous community**				0.622		
No	74 (96.1)	69 (98.6)	Reference			
Yes	3 (3.90)	1 (1.43)	2.56 (0.29–74.6)			

Anti-*St =* anti- *S*. *stercoralis* antibodies

## Discussion

This is the first study that concomitantly assessed anti-*S*. *stercoralis* antibodies and associated risk factors for *S*. *stercoralis* in indigenous populations and their correspondent assistance healthcare professionals with unknown seroprevalence. Indigenous populations are considered one of the most exposed populations to soil-transmitted helminths due to poverty and lack of sanitary conditions [35,36], particularly strongyloidiasis [37]. Brazilian indigenous populations were investigated for seroprevalence and associated risk factors, and we found a relatively high prevalence of strongyloidiasis (37.6%; 174/463; CI 95%: 33.3–42.1) using an ELISA test for anti-IgG against *S*. *stercoralis*. Our results were slightly higher than that reported for adult people living in rural communities of India, where 33.0% (768/2351) were seropositive by ELISA [16]. A review has shown that seroprevalence of *S*. *stercoralis* by immunofluorescence antibody test and ELISA was between 21.7% and 29.2% in the Brazilian general population [38].

Soil-transmitted helminthiases have been ranked among the most important neglected tropical diseases in morbidity [39], particularly affecting the poorest and most vulnerable communities, mostly in tropical and subtropical areas, including Brazilian indigenous populations [23,40]. According to the Center for Disease Control and Prevention (CDC), the main parasitic species infecting people, with respective prevalences, have been roundworms (*Ascaris lumbricoides*), approximately 807–1,121 million; whipworms (*Trichuris trichiura*), approximately 604–795 million; and hookworms (*Necator americanus* and *Ancylostoma duodenale*), approximately 576–740 million worldwide [2]. A meta-analysis focused on soil transmitted helminthiasis in Australian indigenous communities has highlighted the use of a wide range of *S*. *stercoralis* methods for detecting infection, with different sensitivities and specificities, resulting in a plausible prevalence underestimation [41]. Other metanalytic study has included 20 articles on strongyloidiasis prevalence of minority indigenous populations of South-East Asia and the Western Pacific Region, revealing an overall 16.8% of serological prevalence, significantly higher (p = 0.004) than 4.1% observed by microscopy [36]. Although the ELISA test has shown different diagnostic values (75.4–91.2% and 94.8–100%, sensitivity and specificity, respectively) depending on the assay [38], the in-house ELISA test for *S*. *stercoralis* used herein presented excellent sensitivity (95.0%) and specificity (97.8%) performance [38].

Our study compared seropositivity for anti- *S*. *stercoralis* in indigenous populations living with different sanitation conditions. A significantly higher seroprevalence (48.8%, *p* = 0.0004) was observed in indigenous communities living in Paraná state with inadequate sanitation, compared with those living in São Paulo state communities with better sanitary facilities and drinking water quality located in (31.6% seroprevalence). In addition, the presence of septic tanks and drinking water from artesian wells were important protective factors, reducing in 57% the risk for *S*. *stercoralis* seropositivity. Similarly, unimproved sources of drinking water (OR = 2.91) and indiscriminate defecation (OR = 2.81) were the most substantial associated risk factors for strongyloidiasis in schoolchildren living in a rural area in Malaysia [42].

Rivers or creeks, the primary sources of drinking water for indigenous persons living in Paraná state, were potentially contaminated by feces with *Strongyloides* spp., similar to observations in Malaysia [42], and may play a pivotal role in strongyloidiasis transmission. Moreover, improper use of latrines, leading to open defecation practices, may increase environmental contamination with human excreta containing *S*. *stercoralis* larvae [43]. As expected, defecation in latrines was a protective factor for strongyloidiasis (OR = 0.6; p = 0.001) in a rural Cambodian population, decreasing cases by 39% when latrines were adopted by all participants [44]. In addition, strongyloidiasis cases in children of Malaysia were reduced by almost half (48.7%) when all individuals avoided indiscriminate or open defecation in rivers and surrounding areas [42]. In our study, we found that 20.2% of cases could be avoided if children were provided with better drinking water sources in their households.

Age and gender have also been described as risk factors for strongyloidiasis in indigenous populations [36,45] and people living in rural areas [16,44,46]. Herein, adults presented a significantly higher prevalence than young or children (<18 years). In the general Brazilian population, *S*. *stercoralis* seroprevalence has been increased with age, associated with immune disability [38]. Among the 5 regions of Brazil, the difference of seroprevalence varied of 3.9% to 7.9%, with short variation between rural urban areas, of 4.8% to 5.0%, respectively [38]. In addition, being male and have daily activities out their nursing homes were associated with higher *S*. *stercoralis* infection in 10/200 (5.0%) elderly individuals of southeastern Brazil, when compared with other enteroparasites studied [47]. Results herein have also shown that indigenous males represented a risk factor (OR = 1.76) for strongyloidiasis, which may be due to agriculture occupation and higher contact with soil containing infective forms of *Strongyloides* spp. [42,48]. The earlier exposure and subsequent co-infection, and engagement of older boys (12–14 years were 1.56 and 1.96 times, respectively at higher risk compared to children aged 6–9 years) were considered the two main factors for explaining age and gender as risk factors for strongyloidiasis in schoolchildren [43]. A follow-up study with schoolchildren living in semi-rural communities of Cambodia revealed high reinfection rates after two years of anthelmintic treatment and significantly higher reinfection (OR = 3.0) in boys [44]. Thus, in addition to poor sanitation and drinking water sources, continuous exposure due to outdoor and farming activities may explain the sex and age-dependent associated risks for seropositivity we observed.

Protective factors for strongyloidiasis reportedly include using footwear to reduce the chance of cutaneous penetration by *S*. *stercoralis* larvae [46,49]. Since health care workers were non-barefoot, and indigenous adults and children were mostly barefoot at the present study, such associated risk (or protective) factor was not assessed. As observed in all the indigenous communities visited, high parasitic *S*. *stercoralis* loads have been associated with earthen floors in households [19]. Transmission of parasitic diseases has been considered to be facilitated by overcrowded living conditions and infrastructure problems, which result in poor sanitation and hygiene [50], and probably were determinant variables for the infections in the population studied. Unexpectedly, educational level and indigenous ethnicity were not associated to strongyloidiasis, probably due to similar group exposure within communities, despite the differences in personal hygiene. As previously described, Paraná state communities lacked water treatment systems with no septic tanks in their households, while communities of São Paulo state had artesian wells for water supply and septic tanks for feces disposal.

Dogs have been indicated as a potential reservoir for zoonotic transmission of *S*. *stercoralis*, carrying and sharing infection with owners [51]. In our study, with 367/463 (79.3%) indigenous dog owners (average of 2.5 dogs per household), we found no association with seropositivity, corroborating results similar to a previous study of schoolchildren in Malaysia [42]. As our study was limited to human seroprevalence, the role of dogs in the *S*. *stercoralis* cycle of indigenous communities should be further investigated.

Although occupational risk for strongyloidiasis has been documented [52–54], the study herein was the first comparative report of *S*. *stercoralis* seroprevalence between the indigenous population and healthcare professionals. As healthcare professional were represented by persons aging 18 years old or above, we have included only the adult population of indigenous persons for such comparison. A slight tendency (but not statistically significant; p = 0.106) between the seroprevalence in adult indigenous persons (56.1%) and healthcare professionals (53.4%) was observed. As the seropositivity odds was 2.9-fold higher in indigenous persons using unimproved source of drinking water, water consumption was likely as a source of infection in healthcare professionals. Nonetheless, 109/147 (74.2%) healthcare workers reported no water consumption in the indigenous community and no association was observed in the statistical analysis. Even so, only three (2.04%) professionals declared the consumption of water collected from artesian well. In addition, infection may result from person-to-person, and soil or personal objects contact during labor activities, but such practices were not part of the questionnaire. The probability of false-positive results was considered low, since the ELISA performed herein has shown 95% of sensitivity and 97.8% of specificity. Thus, full assessment of healthcare professional labouring during working time in indigenous communities should be further investigated.

An intervention study on indigenous adult populations in Northern Australia found that transmission may continue in endemic areas with inadequate sanitation conditions. Also, intersectoral government collaboration was crucial to interrupt transmission at the community level, and to ensure that disadvantaged communities were not neglected [55]. Thus, to ensure effective intervention herein, the SDIH–Seashore South was immediately informed of the individual results, along with a warning about workers’ health risks and recommendations for improvements of drinking water supply and sanitary infrastructure, despite potential cultural issues.

For limitations, associated risk factors tested herein were based on epidemiological questionnaires, responded by interview with indigenous persons and healthcare professionals at their own knowledge and will for actual answer. Further, indigenous persons were preventively dewormed twice per year with albendazole (May and November) to reduce the risks of helminth infection, following the SSIH protocol. Although Albendazole has been extensively used in massive programs for the control of soil-transmitted helminths [56], this drug has been considered a second-line treatment for *S*. *stercoralis* infection [5]. Herein, indigenous persons may have refused, simulated intake,’ or discharged the anti-helminthics, which could have caused resistance over time, or reduced but not eradicated the chances of by *S*. *stercoralis* infection. It has been stated that mass deworming may be insufficient to eradicate infection in highly risk areas with optimal conditions for *Strongyloides* development [3], as observed in a population of schoolchildren underwent to a two-years- anthelmintic-protocol treatment, in Cambodia [44]. Herein, the second-line treatment with Albendazole has been provided to indigenous population for the last six years. Hence, the deworming program may have contributed to reduce the infection and consequently the seropositivity in younger indigenous. On the other hand, continuous infection or autoinfection may have contributed to the persistence of antibodies in adults. As serological diagnosis by IgG-ELISA may not be very useful in differentiating active and past infections [57], infection of adult indigenous evaluated herein could be either acute or chronic strongyloidiasis.

Finally, a One Health approach may contribute to understanding the mechanisms of strongyloidiasis exposure, including environmental, animal, and human components that pave the way for an interdisciplinary effort to deal with such an important and neglected parasitic disease in indigenous communities.

## Conclusion

This study found a high seroprevalence to *S*. *stercoralis* in indigenous communities of Brazil along with an even higher rate among healthcare professionals. This was particularly true for communities lacking good sanitation conditions, serving as a warning for potential public health concerns of strongyloidiasis in such populations.

## Supporting information

S1 Table Locations and coordinates of Indigenous Communities in Paraná and São Paulo states, including the total and sampled populations. (DOCX)

S2 Table Content for assessing the potential exposure to strongyloidiasis. (DOCX)

S1 Fig Receiver operating characteristic (ROC) curve assessing the accuracy of the multivariate logistic regression model for predicting seropositivity for anti- S. stercoralis antibodies in indigenous populations of southern/southeastern Brazil (top; area under curve (AUC): 0.68; 95% CI: 61.1–71.7). (DOCX)
