# Supplementary material for: Seroprevalence and associated risk factors of strongyloidiasis in indigenous communities and healthcare professionals from Brazil
Source: PLoS Negl Trop Dis. 2023 Apr 27;17(4):e0011283. doi: 10.1371/journal.pntd.0011283 (PMC10168564; doi:10.1371/journal.pntd.0011283)
Supplement: S1 Table — (DOCX) [file pntd.0011283.s001.docx]

**S1 Table.** Locations and coordinates of Indigenous Communities in Paraná and São Paulo state, including the total and sampled populations.

| Indigenous  Community | Coordinates | Total | Sampled | % | Ethnicity | State |
| --- | --- | --- | --- | --- | --- | --- |
| Tekoa Pidoty | 25°31'10.34"S 48°28'06.78"O | 40 | 22 | 55.0 | Guarani | Paraná |
| Kuaray haxa | 25°18'40.65"S  48°18'9.14"O | 25 | 18 | 72.0 | Guarani | Paraná |
| Araça’í | 25°29'23.31"S  49° 0'11.57"O | 90 | 72 | 80.0 | Guarani, Kaingang | Paraná |
| Tupã  Nhe’e Kretã | 25°36'56.21"S 48°56'13.05"O | 30 | 29 | 96.7 | Guarani, Kaingang | Paraná |
| Guaviraty | 25°36'25.35"S 48°26'36.33"O | 39 | 21 | 53.8 | Guarani | Paraná |
| Kopenoty | 22°15’58.20’’S 49°21’00.95’’O | 245 | 125 | 51.0 | Guarani, Kaingang, Terena | São Paulo |
| Tereguá | 22°15'55.31"S 49°20'53.34"O | 127 | 47 | 37.0 | Terena, Guarani | São Paulo |
| Ekeruá | 22°16’28.05’’S 49°22’21.95’’O | 159 | 56 | 35.2 | Guarani, Terena | São Paulo |
| Nimuendaju | 22°17’30.81’’S 49°22’41.03’’O | 100 | 73 | 73.0 | Guarani, Terena | São Paulo |
