# Supplementary material for: Seroprevalence and associated risk factors of strongyloidiasis in indigenous communities and healthcare professionals from Brazil
Source: PLoS Negl Trop Dis. 2023 Apr 27;17(4):e0011283. doi: 10.1371/journal.pntd.0011283 (PMC10168564; doi:10.1371/journal.pntd.0011283)
Supplement: S2 Table — (DOCX) [file pntd.0011283.s002.docx]

**S2 Table.** Content for assessing the potential exposure to strongyloidiasis.

| Topics | Gathered information |
| --- | --- |
| Socio-economic and demographic  characteristics | gender, age, indigenous community, local of community, educational level, ethnicity. |
| Information on domestic animals | dog ownership. |
| Information on drinking water | source of drinking water |
| Sanitary facilities | local of feces disposal |
