# Supplementary material for: Seroprevalence and associated risk factors of strongyloidiasis in indigenous communities and healthcare professionals from Brazil
Source: PLoS Negl Trop Dis. 2023 Apr 27;17(4):e0011283. doi: 10.1371/journal.pntd.0011283 (PMC10168564; doi:10.1371/journal.pntd.0011283)
Supplement: S1 Fig — (DOCX) [file pntd.0011283.s003.docx]

**
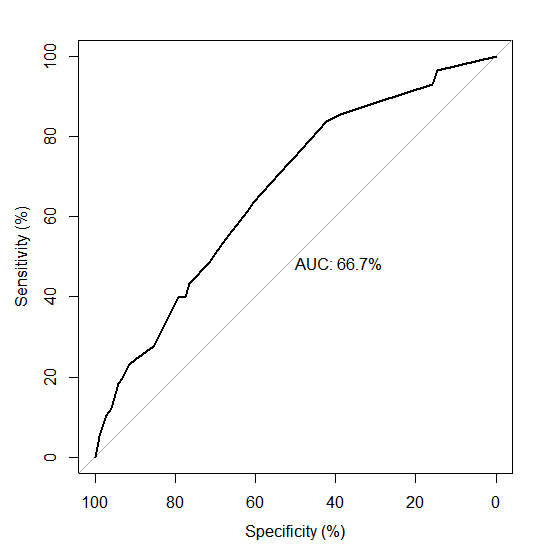
**

**Supplementary Fig 1**- Receiver operating characteristic (ROC) curve assessing the accuracy of the multivariate logistic regression model for predicting seropositivity for anti- *S. stercoralis* antibodies in indigenous populations of southern/southeastern Brazil (top; area under curve (AUC): 0.68; 95% CI: 61.1-71.7). (DOCX)
